# Репликативные и биохимические механизмы старения у женщин с синдромом Тернера

**DOI:** 10.14341/probl13256

**Published:** 2024-01-24

**Authors:** Р. К. Михеев, Е. Н. Андреева, О. Р. Григорян, Е. В. Шереметьева, М. С. Панкратова, Е. В. Логинова

**Affiliations:** Национальный медицинский исследовательский центр эндокринологии; Национальный медицинский исследовательский центр эндокринологии; Московский государственный медико-стоматологический университет имени А.И. Евдокимова; Национальный медицинский исследовательский центр эндокринологии; Национальный медицинский исследовательский центр эндокринологии; Национальный медицинский исследовательский центр эндокринологии; Российский университет дружбы народов

**Keywords:** синдром Тернера, преждевременная недостаточность яичников, кариотип, теломеры, биохимический анализ

## Abstract

**ОБОСНОВАНИЕ:**

ОБОСНОВАНИЕ. В 2025 г. исполняется 100 лет со времени публикации первого в мире описания необычного симптомокомплекса, по современным данным, обусловленного геномными аномалиями и дисгенезией гонад с исходом в гипергонадотропный гипогонадизм, — синдрома Тернера. Тотальный эстрогенный дефицит запускает у пациенток комбинацию сочетанной патологии, что, в свою очередь, указывает на необходимость персонализированной оценки маркеров репликативного клеточного старения у данной категории пациенток.

**ЦЕЛЬ:**

ЦЕЛЬ. Изучить особенности маркеров репликативного клеточного старения (длина теломер лейкоцитов) и биохимических показателей (липидный профиль, кальциево-фосфорный обмен, функциональная активность щитовидной железы, маркеры цитолиза и холестаза, углеводный обмен, азотистый обмен, электролиты, фолликулостимулирующий гормон (ФСГ)) у женщин с синдромом Тернера.

**МАТЕРИАЛЫ И МЕТОДЫ:**

МАТЕРИАЛЫ И МЕТОДЫ. Исследование проведено на базе ФГБУ «НМИЦ эндокринологии» Минздрава России совместно с МНОЦ «МГУ им. М.В. Ломоносова» в период с 10.01.2021 г. по 01.08.2022 г. В одномоментном сравнительном исследовании приняли участие 26 женщин (13–40 лет) с неятрогенным гипергонадотропным гипогонадизмом в исходе синдрома Тернера (45,X0; 45,X/46,XX; 45,X/46,X,r(X)), 26 женщин с преждевременной недостаточностью яичников (ПНЯ) (18–39 лет) и 24 здоровые женщины репродуктивного возраста (15–49 лет). Пациенткам проведен лабораторный генетический (длина теломер лейкоцитов), биохимический анализы крови (гликемия венозной плазмы натощак, мочевина, креатинин, общий/прямой билирубин, аланинаминотрансфераза, аспартатаминотрансфераза, гамма-глутамилтранспептидаза, триглицериды, общий холестерин (ХС), ХС липопротеидов высокой и низкой плотности, общий/ионизированный кальций, фосфор, витамин D, натрий/калий/хлориды, ФСГ, гликированный гемоглобин). Экстракция ДНК — набором Qiagen DNA blood mini kit (Германия). Антропометрическое исследование — длина тела, масса тела. Оценка длины теломер лейкоцитов — методом полимеразной цепной реакции в реальном времени (алгоритм Flowfish). Программа IBM SPSS Statistics (version 26,0 for Windows).

**РЕЗУЛЬТАТЫ:**

РЕЗУЛЬТАТЫ. 1. Женщины с синдромом Тернера имеют достоверно более низкую среднюю длину теломер (8,22 кБ [6,63–9,30]), чем пациентки с ПНЯ (10,34 кБ [8,41–13,08]), p<0,001, и здоровые пациентки репродуктивного возраста (10,77 кБ [9,95–13,16], р>0,05).

**ЗАКЛЮЧЕНИЕ:**

ЗАКЛЮЧЕНИЕ. Синдром Тернера является серьезным генетическим заболеванием, приводящим не только к первичному бесплодию, но и снижению качества/продолжительности жизни.

## Abstract (en)

**BACKGROUND:**

BACKGROUND: 2025 is going to be the 100th anniversary of the first historical description of Turner syndrome — complex of  genomic abnormalities, congenital gonadal disruption and hypergonadotropic hypogonadism. Total estrogenic deficiency triggers development of age-related comorbidities. There is no doubt that personalized search for replicative markers of cellular aging among females with Turner syndrome is needed.

**AIM:**

AIM: To evaluate features of replicative (telomere length) and biochemical (lipid profile, calcium-phosphate album, thyroid hormones, markers cytolysis and cholestasis, carbohydrate metabolism, nitrogenic metabolism, electrolytes, FSH) markers among females with Turner syndrome.

**MATERIALS AND METHODS:**

MATERIALS AND METHODS. Research has been provided in collaboration between Endocrinology Research Centre of the Russian Ministry of Health and Lomonosov Moscow State University Medical Research and Educational Centre in the period since 10.01.2021 until 01.08.2022. Females with non-iatrogenic hypergonadotropic hypogonadism caused by Turner syndrome (45,X0; 45,X/46,XX; 45,X/46,X,r(X); 13–40 y.o.; n=26) and primary ovarian insufficiency (18–39 нyears=26); healthy females of reproductive age (15–49 y.o.; n=24). Patients have undergone laboratory genetic (leucocyte telomere length), biochemical (fasting glycaemia, urea, creatinine, common/conjugated bilirubin, ALT, AST, gamma-glutamyl transferase, triglycerides, HDL-P, LDL-P, common cholesterol, common/ionized calcium, phosphate, vitamin D, sodium/potassium/chlorides, FSH, HbA1c) analyses. Body measurements — body mass, body height. DNA extraction — provided with Qiagen DNA blood mini kit (Germany). Leukocyte telomere length — with real-time polymerase chain reaction PCR (Flow-fish). Soft program IBM SPSS Statistics (version 26,0 for Windows).

**RESULTS:**

RESULTS. 1. Females with Turner syndrome have significantly lower mean telomere length (8,22 kB [6,63–9,30]) than with primary ovarian insufficiency (10, 34 кБ [8,41–13,08], p<0,001) and healthy reproductive age females (10,77 kB [9,95–13,16], р>0,05).

**2:**

2. Telomere length correlates directly and significantly with longevity of menopausal hormonal therapy among females with primary ovarian insufficiency (ρ = 505; p<0,001).

**3:**

3. Patients with Turner syndrome are inclined to vitamin D deficiency (р<0,001), dyslipidemia (р=0,01); increase of levels of aminotransferases, cholestasis markers, phosphate and FSH (р<0,001).

**CONCLUSION:**

CONCLUSION. Turner syndrome is serious genetic disease that leads not only to infertility but to significant decrease of quality/life longevity out of “healthy aging” conception.

## ОБОСНОВАНИЕ

За прошедшее столетие с момента первого описания профессором Н.А. Шерешевским внешних проявлений симптомокомплекса, включающего низкорослость, отсутствие вторичных половых признаков, короткую шею с крыловидными складками, низкий рост волос, микрогнатию, высокое небо и гипертелоризм сосков [1], клиницистами и цитогенетиками многих стран были определены фундаментальные вехи развития синдрома Тернера (СТ). В 1938 г. американский эндокринолог и по совместительству сотрудник Медицинского колледжа Университета Оклахомы (г. Оклахома-Сити, штат Оклахома, США) Генри Тернер (1892–1970) дополнил данный симптомокомплекс вальгусной деформацией локтевых суставов и дисгенезией яичников, благодаря чему его фамилия навсегда запечатлена в истории [2]. Таким образом, успехи советской и американской эндокринологических школ ознаменовали свершение первого исторического (клинико-описательного) этапа изучения данного синдрома. Принципиально новый, цитогенетический, этап, продолжающийся в наши дни, начался с сенсационной статьи руководителя лаборатории Medical Research Council (Харуэлл, графство Оксфордшир, Великобритания) Чарльза Эдмунда Форда (1912–1999) [3], который впервые определил моносомию по Х-хромосоме (45,Х0) как первопричину формирования классического варианта СТ. Нельзя забывать, что многогранность, мозаичность и нередкая стертость клинических проявлений (всего — около 60 фенотипических вариантов) зачастую приводят к поздней и случайной диагностике; например варианты 46,X,i(Xq) + мозаики с линиями клеток i(Xq); 46,X,del (Xq) + мозаики с линиями клеток del(Xq); 45X0/46XY; 45X/46XX) [4]. Кроме того, в середине 2000-х годов D. Keefe и соавт. [5] в ходе исследований in vitro наблюдали процесс формирования хромосомных/геномных аберраций, включавший в себя нарушение переплетения двух хроматид (хиазм) в профазе мейоза, аномалию веретена деления и укорочение концевых участков хромосом — теломер.

Теломеры (от др.-греч. τέλος — конец + μέρος — часть) служат концевыми структурами ДНК, состоящими из тандемных повторов нуклеотидных последовательностей TTAGGG на 3’-конце, в частности TPP1 [6]. Стабильность ДНК теломер поддерживается ревертазно-активным высокорегулируемым клеточным ферментом теломеразой [7], защищающей хромосомную ДНК от различных повреждений в течение всего клеточного цикла. По данным L. Gaydosh и соавт. (2020), статистически значимое протективное влияние на теломеры было обнаружено у эстрогенов in vitro [8], что подтверждается достижением более выраженной длины теломер к концу пубертата у женщин по сравнению с мужчинами (исходный показатель при рождении: 7,01±0,03 kb против 6,87±0,04, наблюдение: 6,79±0,03 против 6,65±0,03, Р=0,005) [9].

Характерная для классического кариотипа 45,X0 внутриутробная дисгенезия гонад, сопровождающаяся апоптозом предшественников половых клеток и заменой на волокнистую соединительную ткань (т.н. «гонадный тяж»), приводит к развитию врожденной преждевременной недостаточности яичников (ПНЯ) и тяжелого неятрогенного гипергонадотропного гипогонадизма [10]. На фоне первичной аменореи у пациенток с СТ также развивается тотальный эстрогенный дефицит, чреватый стремительным развитием заболеваний, ассоциированных со старением (атеросклероз, артериальная гипертония, дислипидемия, инсулинорезистентность, сердечно-сосудистые, неврологические, костно-мышечные заболевания) [11]. Эстрогенный дефицит при СТ — прямое показание к инициации пожизненной заместительной гормональной терапии (ЗГТ) [12].

## ЦЕЛЬ ИССЛЕДОВАНИЯ

Изучить особенности маркеров репликативного клеточного старения (длина теломер лейкоцитов) и биохимических показателей (липидный профиль, кальциево-фосфорный обмен, функциональная активность щитовидной железы, маркеры цитолиза и холестаза, углеводный обмен, азотистый обмен, электролиты, фолликулостимулирующий гормон (ФСГ)) у женщин с СТ.

## МАТЕРИАЛЫ И МЕТОДЫ

## Место и время проведения исследования

Исследование проведено на базе ФГБУ «НМИЦ эндокринологии» Минздрава России совместно с МНОЦ «МГУ им. М.В. Ломоносова» в период с 10.01.2021 г. по 01.08.2022 г.

## Дизайн исследования

В одномоментном сравнительном исследовании всего приняли участие 76 женщин, из них:

1.26 женщин (13–40 лет) с СТ (45,X0; 45,X/46,XX; 45,X/46,X,r(X)), получавших ЗГТ в циклическом режиме с дозой эстрогенового компонента 2 мг и дидрогестерона 10 мг.

2.26 женщин (18–39 лет) с преждевременной недостаточностью яичников (ПНЯ), получавших ЗГТ в циклическом режиме с дозой эстрогенового компонента 2 мг и дидрогестерона 10 мг.

3.24 здоровые женщины репродуктивного возраста.

## Изучаемые популяции пациентов

Пациентки с СТ, получающие заместительную терапию половыми стероидами.

Критерии включения: пациентки женского пола паспортного возраста 13–40 лет; кариотип 45,X0; 45,X/46,XX; 45,X/46,X,r(X) — по данным медицинской документации. В анамнезе — факт получения ЗГТ в циклическом режиме с дозой эстрогенового компонента 2 мг и дидрогестерона 10 мг в течение не менее 5 лет. Пациентки (при недостижении возраста 18 лет — их родители) подписывали информированное согласие на проведение обследования и консультирование.

Критерии невключения.

1.Кариотип 45,XY.

2.После перенесенной химиотерапии.

3.После перенесенной лучевой терапии.

4.После комбинированного лечения из вышеперечисленных выше методов.

5.Наличие сопутствующей патологии в анамнезе:

5.1.нарушения функции щитовидной железы;

5.2.наличие официально задокументированных психических расстройств;

5.3.наличие официально задокументированных злокачественных новообразований;

5.4.нарушения углеводного обмена (нарушенная толерантность к глюкозе, нарушенная гликемия венозной плазмы натощак, сахарный диабет 1 и 2);

5.5.сердечно-сосудистые заболевания (ишемическая болезнь сердца (ИБС), острое нарушение мозгового кровообращения (ОНМК), тромбоэмболия легочной артерии (ТЭЛА)).

Способ формирования выборки — сплошной.

## Пациентки с установленным диагнозом «Преждевременная недостаточность яичников», получающие заместительную терапию половыми стероидами >5 лет.

Критерии включения: пациентки женского пола паспортного возраста 18–39 лет, находящиеся в состоянии аменореи длительностью не менее 5 лет; подтвержденный медицинской документацией факт получения ЗГТ в циклическом режиме с дозой эстрогенового компонента эстрадиола 2 мг и дидрогестерона 10 мг в течение не менее 5 лет. Все пациентки подписывали информированное согласие на проведение обследования и консультирование.

Критерии невключения.

1.Наличие ятрогенной менопаузы в анамнезе:

1.1.после перенесенных хирургических вмешательств (кастрации);

1.2.после перенесенной химиотерапии;

1.3.после перенесенной лучевой терапии;

1.4.после комбинированного лечения из вышеперечисленных выше методов.

2.Наличие сопутствующей патологии в анамнезе:

2.1.генетическая патология репродуктивной системы (СТ, кариотип 45,X0/XY);

2.2.эстроген-зависимые заболевания и патологические состояния (гиперпластические процессы эндометрия, миома матки, все формы эндометриоза);

2.3.нарушения функции щитовидной железы;

2.4.наличие официально задокументированных психических расстройств;

2.5.наличие официально задокументированных злокачественных новообразований;

2.6.нарушения углеводного обмена (нарушенная толерантность к глюкозе, нарушенная гликемия венозной плазмы натощак, сахарный диабет 1 и 2);

2.7.сердечно-сосудистые заболевания (ИБС, ОНМК, ТЭЛА).

3.Другие физиологические состояния репродуктивной системы:

3.1.беременность;

3.2.период грудного вскармливания.

Способ формирования выборки — произвольный.

## Здоровые женщины репродуктивного возраста без заболеваний репродуктивной системы.

Критерии включения: пациентки женского пола с сохраненным менструальным циклом; уровень ФСГ в фолликулярную фазу — в пределах 2,0–11,6 МЕ/л. Все пациентки подписывали информированное согласие на проведение обследования и консультирование.

Критерии невключения.

1.Наличие физиологической менопаузы в анамнезе.

2.Наличие ятрогенной менопаузы в анамнезе:

2.1.после перенесенных хирургических вмешательств (кастрации);

2.2.после перенесенной химиотерапии;

2.3.после перенесенной лучевой терапии;

2.4.после комбинированного лечения из вышеперечисленных выше методов.

3.Наличие сопутствующей патологии в анамнезе:

3.1.генетическая патология репродуктивной системы (СТ, кариотип 45,X0/XY);

3.2.наличие аутоиммунной менопаузы в анамнезе (в исходе первичной недостаточности яичников);

3.3.эстроген-зависимые заболевания и патологические состояния (гиперплазия эндометрия, миома матки, все формы эндометриоза);

3.4.нарушения функции щитовидной железы;

3.5.наличие официально задокументированных психических расстройств;

3.6.наличие официально задокументированных злокачественных новообразований;

3.7.нарушения углеводного обмена (нарушенная толерантность к глюкозе, нарушенная гликемия венозной плазмы натощак, сахарный ­диабет 1 и 2);

3.8.сердечно-сосудистые заболевания (ИБС, ОНМК, ТЭЛА).

4.Другие физиологические состояния репродуктивной системы:

4.1.беременность;

4.2.период грудного вскармливания.

Способ формирования выборки — произвольный.

## Описание вмешательства

Пациенткам проведены лабораторный генетический (длина теломер лейкоцитов), биохимический анализы.

Экстракция ДНК проведена набором Qiagen DNA blood mini kit (Германия).

Антропометрическое исследование — длина тела, масса тела.

Оценка длины теломер лейкоцитов — методом полимеразной цепной реакции (ПЦР) в реальном времени (алгоритм Flow-fish).

## Дизайн исследования

Обсервационное одномоментное сравнительное ретроспективное исследование.

## Статистический анализ

Статистическая обработка данных выполнена с помощью программы IBM SPSS Statistics (version 26,0 for Windows).

Категориальные переменные представлены в виде абсолютных и относительных частот. Количественные показатели оценивались на предмет соответствия нормальному распределению с помощью критерия Шапиро–Уилка. В случае отсутствия нормального распределения количественные данные описывались с помощью медианы (Me) и нижнего и верхнего квартилей (Q¹–Q³). При нормальном распределении — средними значениями и стандартным отклонением. Сравнение двух групп по количественному показателю, распределение которого отличалось от нормального, выполнялось с помощью U-критерия Манна–­Уитни. При нормальном распределении использовался t-критерий Стьюдента. Анализ многопольных таблиц сопряженности выполнялся с помощью точного критерия Фишера. Корреляция между переменными была проверена с помощью корреляционного анализа по методу ρ Спирмена. Прогностическая модель, характеризующая зависимость количественной переменной от факторов, разрабатывалась с помощью метода линейной регрессии.

## Этическая экспертиза

Протокол исследования был одобрен этическим комитетом НМИЦ эндокринологии (№11 от 22.07.2021).

## РЕЗУЛЬТАТЫ

Клинико-лабораторные (биохимические и генетические) данные пациенток с неятрогенным гипергонадотропным гипогонадизмом в исходе СТ (кариотип 45,X0) представлены в табл. 1.

**Table:** Таблица 1. Данные пациенток с синдромом Тернера (45,X0) и ПНЯ Данные представлены в виде средних и стандартного отклонения (#) и/или медианы и интерквартильного размаха (##), а также относительных частот (%).* — различия показателей статистически значимы (p<0,05).Алгоритм расчета данных — см. раздел «Статистический анализ».Примечание: ИМТ — индекс массы тела, ТГ — триглицериды, ХС ЛПВП — холестерин липопротеидов высокой плотности, ОХС — общий холестерин, ХС ЛПНП — холестерин липопротеинов низкой плотности, АЛТ — аланинаминотрансфераза, АСТ — аспартатаминотрансфераза, ГГТ — гамма-глутамилтранспептидаза, ФСГ — фолликулостимулирующий гормон, ТТГ — тиреотропный гормон, HbA1c — glycated hemoglobin — гликированный гемоглобин, кБ — килобаза (число тысяч пар азотистых оснований).

Параметр	Женщины с СТ(n=26)	Группа 2ПНЯ (+)(n=26)	р
Возраст, лет #	22,0±7,9	33,7±7,2	<0,001*
Возраст, лет ##	20,5 [ 15,0–27,75]	35,5 [ 28,0–39,0]	<0,001*
ИМТ, кг/м² #	24,0±4,7	23,5±4,3	0,9
ИМТ, кг/м² ##	24,1 [ 19,3–27,4]	21,8 [ 19,9–27,8]	0,9
Сахарный диабет/предиабет, %	23,0	3,8	0,09
Гипотиреоз, %	11,5	15,4	1,0
Альбумин, г/л#	43,9±2,2	44,4±2,5	0,4
Мочевина, ммоль/л #	5,1±1,4	6,7±2,0	0,016*
Мочевина, ммоль/л##	4,7 [ 3,8–6,5]	6,3 [ 5,0–7,4]	0,016*
Билирубин общий, ммоль/л#	8,8±4,9	9,5±7,2	0,1
Билирубин прямой, ммоль/л#	3,9±1,8	4,4±2,2	0,3
Глюкоза натощак, ммоль/л#	4,9±0,8	4,8±0,7	0,5
ТГ, ммоль/л#	1,12±0,41	0,92±1,11	0,009*
ТГ, ммоль/л##	1,10 [ 0,89–1,66]	0,84 [ 0,65–1,03]	0,009*
ХС ЛПВП, ммоль/л#	1,31±0,35	1,74±0,55	0,004*
ХС ЛПВП, ммоль/л##	1,26 [ 1,01–1,96]	1,84 [ 1,66–2,12]	0,004*
ОХС, ммоль/л##	5,1 [ 4,5–5,4]	4,7 [ 4,3–5,1]	0,3
ХС ЛПНП, ммоль/л#	3,22±0,88	2,67±0,71	0,01*
Кальций общий, ммоль/л#	2,34±0,09	2,33±0,08	0,9
Кальций ионизированный, ммоль/л#	1,10±0,04	1,09±0,04	0,3
Фосфор, ммоль/л#	1,46±0,22	1,18±0,13	<0,001*
АЛТ, ЕД/л#	28,67±10,214	13,6±3,5	<0,001*
АЛТ, ЕД/л##	29,0 [ 21,0–35,0]	13,0 [ 10,0–15,0]	<0,001*
АСТ, ЕД/л#	24,38±5,686	16,5±2,3	<0,001*
АСТ, ЕД/л##	22,5 [ 19,0–32,5]	16,5 [ 15,0–19,0]	<0,001*
ГГТ, ЕД/л#	34,81±22,72	18,3±4,28	<0,001*
ГГТ, Ед/л##	27,0 [ 18,5–39,5]	16,0 [ 13,0–19,5]	<0,001*
25(ОН)вит. D, нг/мл#	16,31±6,3	34,06±13,2	<0,001*
25(ОН)вит. D, нг/мл##	15,05 [ 12,05–23,77]	27,80 [ 23,15–41,10]	<0,001*
Натрий, ммоль/л#	139,0±3,0	137,9±2,9	0,1
Калий, ммоль/л#	4,0±1,2	4,8±1,1	0,002*
Хлориды, ммоль/л#	103,0±1,8	104,5±1,4	0,06
ФСГ, мМЕд/л##	107,0 [ 103,2–110,5]	92,0 [ 91,0–95,0]	<0,001*
ТТГ, мЕд/л#	2,6±0,73	3,6±1,49	0,7
ТТГ, мЕд/л##	2,25 [ 1,84–2,97]	2,05 [ 1,32–3,51]	0,7
HbA1c, %#	5,48±0,43	5,45±0,38	0,7
Длина теломер (кБ) ## Min,maх	8,22 [ 6,63–9,30] 5,61–12,40	10,34 [ 8,41–13,08] 6,71–14,40	<0,001*

С целью более наглядного сопоставления длин теломер у исследуемых групп (СТ/ПНЯ) в качестве референсных показателей мы также привели аналогичные показатели здоровых женщин репродуктивного возраста (контрольная группа, n=24). Медиана возраста в данной группе составила 36,5 [ 28,5–40,5] года, ИМТ — 21,8 [ 20,2–27,2] кг/м², ФСГ — 5,8 [ 4,6–8,6] мМЕд/л, длина теломер — 10,77 кБ [ 9,95–13,16], минимум — 8,78, максимум — 14,91.

Все пациентки с ПНЯ (n=26) с момента установления данного диагноза получали ЗГТ, средний срок приема составил 3,0 [ 2,0–5,5] года.

Нами был проведен корреляционный анализ взаимосвязи длины теломер и длительности приема ЗГТ у женщин с ПНЯ. Установлена прямая статистически значимая корреляционная связь умеренной силы (ρ=505; p<0,001).

Исходя из вышеуказанного анализа данных, наблюдаемую зависимость такого маркера клеточного старения, как длина теломер, от количества лет приема ЗГТ можно описать прогностической моделью (уравнением) линейной регрессии:

ZДлина теломер оконч, кБ = 0,216 × Xприем ЗГТ, лет + YДлина теломер начальная, кБ + 9,49

При увеличении приема ЗГТ на 1 год следует ожидать увеличения длины теломер на 0,216 (p <0,001) кБ.

Подтверждение данной прогностической модели хорошо прослеживается у пациенток с ПНЯ, длина теломер которых (на фоне приема ЗГТ) оказалась приближена к референтным нормам здоровых женщин (10,34 [ 8,41–13,08] у женщин с ПНЯ и 10,77 [ 9,95–13,16] у здоровых женщин, р>0,05).

У пациенток с СТ по сравнению с ПНЯ, согласно табл. 1, отмечается статистически значимая тенденция к более низкому уровню липопротеинов высокой плотности (р=0,004), витамина D (p<0,001) при более высоком уровне фосфора сыворотки вплоть до верхней границы референсных значений (p<0,001); более высокому уровню трансаминаз, лабораторным показателям холестаза (ГГТ), триглицеридов, ФСГ (р<0,001), липопротеинов низкой плотности (р=0,01). Также была проведена стратификация показателей длины теломер в исследуемых группах по возрастному критерию (см. табл. 2)

**Table:** Таблица 2. Длина теломер в возрастных группах исследуемых * — различия статистически значимы.

Возрастные группы, длина теломер	Исследуемые группы	р
СТ (n=26)	ПНЯ (n=26)
13–18 лет % (n) Длина теломер Ме [ 25–75%]	42,3 (11) 8,20 [ 6,28–8,74]	3,8 (1) 10,00 [ 10,00–10,00]	<0,001*
19–29 лет % (n) Длина теломер Ме [ 25–75%]	34,6 (9) 8,40 [ 6,27–9,77]	26,9 (7) 9,25 [ 8,40–14,40]	<0,001*
30–39 лет % (n) Длина теломер Ме [ 25–75%]	23,1 (6) 8,11 [ 7,22–10,35]	53,9 (14) 10,37 [ 8,09–11,56]	<0,001*
40–49 лет % (n) Длина теломер Ме [ 25–75%]	0 –	15,4 (4) 11,74 [ 9,90–13,93]	–

## ОБСУЖДЕНИЕ

СТ — генетическое заболевание, которое выявляется у 50 из 100 000 новорожденных девочек и отрицательно влияет на прогноз продолжительности и качества жизни [13]. Соматический статус при данном заболевании разнообразен, всегда имеет коварный и необратимый характер за счет сопутствующих кардиоваскулярных (двухстворчатый аортальный клапан, коарктация аорты, удлинение интервала Q–T), ортопедических (аномальная низкорослость, сколиотические деформации позвоночника), оториноларингологических (тугоухость вплоть до глухоты), патологий, а также нарушений мочевыводящей (почечные мальформации), репродуктивной (первичная аменорея, в ряде случаев — задержка полового развития), эндокринной (метаболический синдром, хронический аутоиммунный тиреоидит, дефицит витамина D, остеопороз) и других систем [14].

## Репрезентативность выборок

В оригинальном исследовании задействован относительно малый объем выборки, что объясняется редкой встречаемостью гипергонадотропного гипогонадизма дисгенетического (СТ) и аутоиммунного характера (ПНЯ) в общей популяции. Кроме того, на итоговый объем выборки серьезным образом повлиял принцип строгого соблюдения критериев включения и (по данным анамнеза и катамнеза) во избежание искажения конечных результатов.

## Сопоставление с другими публикациями

Еще задолго до внедрения современных принципов научной методологии проводились исследования, посвященные сердечно-сосудистым рискам у пациенток с СТ. Согласно данным многолетнего (с 1977 по 2017 г.) клинического исследования на базе кардиохирургической клиники Mayo Clinic Rochester, из группы, состоящей из 281 пациентки, отобраны 51 пациентка (средний возраст — 28 лет, вариабельность от 8 до 41), прошедшая по крайней мере 1 плановое кардиохирургическое вмешательство [15]. Данное исследование наглядно иллюстрирует факт наличия у пациенток с СТ (45,X0) обширного спектра сочетанных терапевтических и кардиохирургических патологий, в т.ч. их комбинаций [15]. Кроме того, наличие СТ ухудшает прогноз пациенток в отношении состояния углеводного обмена: по данным S.M. Davis и M.E. Geffner (2019), при данной нозологии риск сахарного диабета 1 и 2 типов в 11,56 и 4,38 раза выше, чем в общей популяции, соответственно [16].

Одной из первых работ по биологии теломер in vivo у лиц с СТ стало исследование М. Kveiborg и соавт. (2001), выполненное в г. Орхус, Дания. Техническая часть включала в себя оценку скорости укорочения длины теломер (RTFL, Telomere Restriction Fragment Length) у пациенток молодой/старшей возрастных групп с СТ (45,X0; n=30) и нормальным кариотипом (46,XX; n=30) [15]. Убедительных статистически значимых данных за ускоренное укорочение теломер в молодой (СТ: 7011±521 vs. контроль: 7285±917 bp, р=0,3) и старшей возрастных группах (TS: 7357±573 vs. контроль: 7221±621 bp, р=0,6) найдено не было [17]. Отсутствие подтверждения первоначальной гипотезы можно объяснить малым объемом выборки (n=60), что, в свою очередь, является логичным следствием временной, финансово-технической ограниченности и трудностей в наборе основной группы.

## Клиническая значимость результатов

ЗГТ половыми стероидами у женщин с гипергонадотропным гипогонадизмом — собирательное понятие, объединяющее фармакологическую компенсацию эстрогенного дефицита как физиологического (менопаузального), так и патологического (ПНЯ, СТ, нарушение формирования пола) генеза. Ее основной задачей является восстановление уровня эстрогенов до показателей репродуктивного периода. В основе данного эмпирического подхода лежит многолетний клинический опыт ведения пациенток с манифестацией возраст-ассоциированных заболеваний в период менопаузы. В свете ориентированности научного сообщества на принципы персонализированной медицины теломеразная теория старения в ближайшей и отдаленной перспективе будет занимать особое место в повестке дня.

## Ограничения исследования

Наиболее вероятным и потенциально устранимым фактором, ограничивающим данное исследование, является относительная узость выборки ввиду высокой финансовой-технической емкости технологий определения длины теломер, доступных на настоящий момент специалистам в Российской Федерации.

## Направления дальнейших исследований

В перспективе мы предполагаем достоверно определить характер влияния ЗГТ половыми стероидами на длину теломер через проведение биопсии (пункции) яичников и последующее определение длины теломер. На сегодняшний день данная идея пока остается трудноосуществимой на практике с этической и финансово-технической точек зрения.

## ЗАКЛЮЧЕНИЕ

Результаты данного исследования имеют научную ценность, являются показательными, но в то же время не исчерпывающими сами по себе. Только при условии проведения двухмоментных слепых плацебо-контролируемых рандомизированных клинических исследований (РКИ) среди обширных выборок пациенток с СТ результаты могут приобрести практическую ценность для специалистов. Проведение РКИ у пациенток с СТ — неотъемлемая часть разработки эффективных мер по персонализированной фармакологической компенсации данной категории больных в будущем.

## ДОПОЛНИТЕЛЬНАЯ ИНФОРМАЦИЯ

Источник финансирования. Исследование проводится в рамках Государственного задания: «Влияние эпигенетических факторов на течение менопаузы у женщин с эндокринопатиями аутоиммунного генеза в рамках формирования модели “здорового старения”», регистрационный номер АААА-121030100033-4.

Конфликт интересов. Авторы декларируют отсутствие явных и потенциальных конфликтов интересов, связанных с публикацией настоящей статьи.

Участие авторов. Все авторы одобрили финальную версию статьи перед публикацией, выразили согласие нести ответственность за все аспекты работы, подразумевающую надлежащее изучение и решение вопросов, связанных с точностью или добросовестностью любой части работы.

## References

[cit1] Dedov I.I., Peterkova V.A., Volevodz N.N. Sindrom Shereshevskogo–Ternera (patogenez, klinika, diagnostika, lechenie). Metodicheskoe posobie dlya vrachei. — M.; 2009.

[cit2] TURNER HENRY H. (2009). A SYNDROME OF INFANTILISM, CONGENITAL WEBBED NECK, AND CUBITUS VALGUS1. Endocrinology.

[cit3] FORD C (2003). A SEX-CHROMOSOME ANOMALY IN A CASE OF GONADAL DYSGENESIS (TURNER'S SYNDROME). The Lancet.

[cit4] Gravholt CH, Andersen NH, Conway GS, et al. Clinical practice guidelines for the care of girls and women with Turner syndrome: proceedings from the 2016 Cincinnati International Turner Syndrome Meeting. Eur J Endocrinol. 2017;177(3):G1-G70. doi: https://doi.org/10.1210/endo-23-5-566.10.1530/EJE-17-0430 28705803

[cit5] Keefe DL, Marquard K, Liu L. The telomere theory of reproductive senescence in women. Curr Opin Obstet Gynecol. 2006;18(3):280-285. doi: https://doi.org/10.1210/endo-23-5-566.10.1097/01.gco.0000193019.05686.49 16735827

[cit6] Liu Baocheng, He Yao, Wang Yaqiang, Song He, Zhou Z. Hong, Feigon Juli (2022). Structure of active human telomerase with telomere shelterin protein TPP1. Nature.

[cit7] Roake CM, Artandi SE. Regulation of human telomerase in homeostasis and disease. Nat Rev Mol Cell Biol. 2020;21(7):384-397. doi: https://doi.org/10.1038/s41580-020-0234-z 32242127 PMC7377944

[cit8] Turner KJ, Vasu V, Griffin DK. Telomere biology and human phenotype. Cells. 2019;8(1):73. doi: https://doi.org/10.1210/endo-23-5-566.10.3390/cells8010073. PMC6356320 30669451

[cit9] Gaydosh Lauren, Mitchell Colter, Notterman Daniel, Schneper Lisa, Brooks-Gunn Jeanne, Wagner Brandon, Koss Kalsea, McLanahan Sara (2021). Demographic and developmental patterns in telomere length across adolescence. Biodemography and Social Biology.

[cit10] Ishizuka Bunpei (2021). Current Understanding of the Etiology, Symptomatology, and Treatment Options in Premature Ovarian Insufficiency (POI). Frontiers in Endocrinology.

[cit11] Gravholt CH, Andersen NH, Conway GS, et al. Clinical practice guidelines for the care of girls and women with Turner syndrome: proceedings from the 2016 Cincinnati International Turner Syndrome Meeting. Eur J Endocrinol. 2017;177(3):G1-G70. doi: https://doi.org/10.1530/EJE-17-0430 28705803

[cit12] Klein Karen O, Rosenfield Robert L, Santen Richard J, Gawlik Aneta M, Backeljauw Philippe F, Gravholt Claus H, Sas Theo C J, Mauras Nelly (2018). Estrogen Replacement in Turner Syndrome: Literature Review and Practical Considerations. The Journal of Clinical Endocrinology & Metabolism.

[cit13] Donadille Bruno, Christin-Maitre Sophie (2020). Heart and Turner syndrome. Annales d'Endocrinologie.

[cit14] Shankar Kikkeri N, Nagalli S. Turner Syndrome. In: StatPearls. Treasure Island (FL): StatPearls Publishing; 2022. 32119508

[cit15] Fuchs Margaret M, Jost Christine Helena Attenhofer, Said Sameh M, Hagler Donald J, Connolly Heidi M, Dearani Joseph A, Egbe Alexander C (2020). Cardiovascular surgery in Turner syndrome - early outcome and long-term follow-up. World Journal of Cardiology.

[cit16] Davis Shanlee M., Geffner Mitchell E. (2019). Cardiometabolic health in Turner syndrome. American Journal of Medical Genetics Part C: Seminars in Medical Genetics.

[cit17] Kveiborg Marie, Gravholt Claus Højbjerg, Kassem Moustapha (2008). Evidence of a normal mean telomere fragment length in patients with Ullrich-Turner syndrome. European Journal of Human Genetics.

